# Risk Prediction in Sexual Health Contexts: Protocol

**DOI:** 10.2196/resprot.2971

**Published:** 2013-12-03

**Authors:** Titilola Falasinnu, Paul Gustafson, Mark Gilbert, Jean Shoveller

**Affiliations:** ^1^School of Population and Public HealthUniversity of British ColumbiaVancouver, BCCanada; ^2^Department of StatisticsUniversity of British ColumbiaVancouver, BCCanada; ^3^Clinical Prevention ServicesBritish Columbia Center for Disease ControlVancouver, BCCanada

**Keywords:** prediction models, Internet-based testing, sexually transmitted infections

## Abstract

**Background:**

In British Columbia (BC), we are developing Get Checked Online (GCO), an Internet-based testing program that provides Web-based access to sexually transmitted infections (STI) testing. Much is still unknown about how to implement risk assessment and recommend tests in Web-based settings. Prediction tools have been shown to successfully increase efficiency and cost-effectiveness of STI case finding in the following settings.

**Objective:**

This project was designed with three main objectives: (1) to derive a risk prediction rule for screening chlamydia and gonorrhea among clients attending two public sexual health clinics between 2000 and 2006 in Vancouver, BC, (2) to assess the temporal generalizability of the prediction rule among more recent visits in the Vancouver clinics (2007-2012), and (3) to assess the geographical generalizability of the rule in seven additional clinics in BC.

**Methods:**

This study is a population-based, cross-sectional analysis of electronic records of visits collected at nine publicly funded STI clinics in BC between 2000 and 2012. We will derive a risk score from the multivariate logistic regression of clinic visit data between 2000 and 2006 at two clinics in Vancouver using newly diagnosed chlamydia and gonorrhea infections as the outcome. The area under the receiver operating characteristic curve (AUC) and the Hosmer-Lemeshow statistic will examine the model’s discrimination and calibration, respectively. We will also examine the sensitivity and proportion of patients that would need to be screened at different cutoffs of the risk score. Temporal and geographical validation will be assessed using patient visit data from more recent visits (2007-2012) at the Vancouver clinics and at clinics in the rest of BC, respectively. Statistical analyses will be performed using SAS, version 9.3.

**Results:**

This is an ongoing research project with initial results expected in 2014.

**Conclusions:**

The results from this research will have important implications for scaling up of Internet-based testing in BC. If a prediction rule with good calibration, discrimination, and high sensitivity to detect infection is found during this project, the prediction rule could be programmed into GCO so that the program offers individualized testing recommendations to clients. Further, the prediction rule could be adapted into educational materials to inform other Web-based content by creating awareness about STI risk factors, which may stimulate health care seeking behavior among individuals accessing the website.

## Introduction

### Getting Checked Online

There has been considerable interest in the adoption of information and communication technology for prioritizing resources in sexually transmitted infections (STI) service delivery [1,2]. In British Columbia (BC), we are developing Get Checked Online (GCO), an Internet-based testing program that provides Web-based access to STI testing [3]. The overall goal of GCO is to reduce barriers to accessing appropriate sexual health services, and ultimately to decrease the overall burden of STI/human immunodeficiency virus (HIV) in BC. Clients accessing GCO will complete a risk assessment module, download a test requisition (if appropriate), provide blood and/or urine specimens at designated specimen collection sites, and retrieve negative results on the Internet or positive results in-person or by telephone [3]. By selectively triaging asymptomatic and other low risk GCO clients for laboratory testing only, the hope is to most efficiently identify infections. Universal screening is not likely to be cost-effective in a population with relatively low STI/HIV prevalence, including the general population of BC. Selective screening based on risk assessment may optimize cost-effectiveness and limit the number of individuals confronted with unnecessary tests [4].

### Implementing Risk Assessment

Much is still unknown about how to implement risk assessment and recommend tests in Web-based settings. In traditional sexual health service settings (such as STI clinics), screening guidelines or recommendations provide clinicians with assistance in distilling and applying the scientific literature to recommend specific STI tests and prioritize patient groups. Prediction tools have been shown to successfully increase efficiency and cost-effectiveness of STI case finding in the following settings-HIV screening [5], Internet-based testing [4,6], and partner notification [7-9]. These tools, broadly termed *clinical prediction rules*, use combinations of risk factors that have been statistically demonstrated to be meaningful predictors to calculate a numerical probability of the presence of a specific condition or likelihood of an outcome [10,11].

### The Methodological Framework

While acknowledging important early initiatives [4-6], the methodology for evaluation of STI prediction rules is not yet as crystallized as the methodologies associated with prediction rules used for chronic disease management (eg, the Framingham risk score for estimating cardiovascular disease). There has also been little discussion of practical considerations, especially issues associated with the formal validity of prediction tools that are particularly salient for STI service delivery. In this study, we describe the methodological framework for using electronic health records to develop and validate a multivariable risk prediction rule among clients attending STI clinics in BC. Specifically, this project was designed with three main objectives: (1) to derive a risk prediction rule for screening chlamydia and gonorrhea among clients attending two public sexual health clinics between 2000 and 2006 in Vancouver, BC, (2) to assess the temporal generalizability of the prediction rule among more recent visits in the Vancouver clinics (2007-2012), and (3) to assess the geographical generalizability of the rule in seven additional clinics in BC.

## Methods

### The Prediction Rule

**Figure 1:**
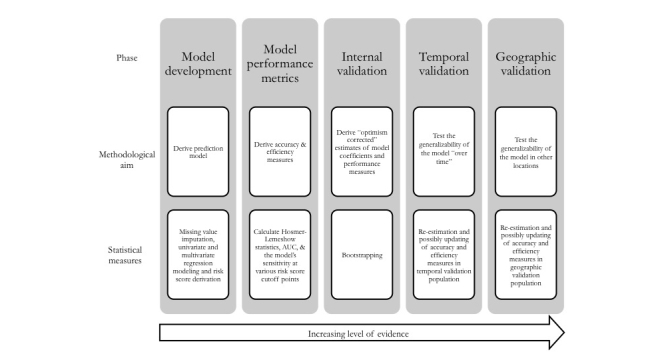
Methodological framework for the derivation and validation of the prediction rule.

Figure 1 shows the methodological framework for the derivation and validation of the prediction rule.

### Study Populations

This study will involve a population-based, cross-sectional analysis of electronic records of patients visits collected at publicly funded STI clinics that offer physical examination and treatment for STIs in BC. Data from each new client consultation between 2000 and 2012 among women and men who have sex with women will be included in this study. This analysis will be limited to asymptomatic clinic visits that are not sexual contacts of known STI cases. Repeat visits within 30 days of a previous clinical visit will also be excluded to avoid including clients receiving confirmatory diagnoses. The prediction rule will be created using the data gathered from the *development population* and the generalizability of the criteria will be tested in the *validation populations*. The *development population* is comprised of patient visits at the 12^th^ Avenue and Bute Street clinics in Vancouver (n=10,471; chlamydia and/or gonorrhea prevalence is, 1.76%). These are low-threshold (free of charge and, if preferred, pseudonymously), outpatient clinics run by the BC Center for Disease Control (BCCDC). They provide STI assessment and management services, including HIV testing, for clients from throughout the Vancouver area. Chlamydia, gonorrhea, syphilis, and HIV tests are offered to all sexually active clients at each clinic visit.

The external validity of the model, known as the performance in different populations (also labeled “generalizability” or “transportability”), will also be tested. Temporal validity is generally considered the first line of generalizability ascertainment. This issue is particularly salient in STI testing because of the shift towards more sensitive diagnostic tests over this time period. The *temporal validation population* will include more recent visits at the Vancouver clinics between 2007 and 2012 (n=15,107; chlamydia and/or gonorrhea prevalence is, 2.23%).

The *geographical validation populations* will include clinic visits in publicly funded sexual health clinics located in the following geographical locations in BC–Penticton, Kelowna, Kamloops, New Westminster, Boundary, Courtenay, and Prince George. The proposed study will analyze computerized records from clients attending these clinics between 2000 and 2012 (n=10,529; chlamydia and/or gonorrhea prevalence is, 5.37%). These public sexual health clinics in BC use the same electronic charting as the BCCDC clinics, thus, the consistent nature of the data collection methods across the clinics allows for the direct comparison of data between individuals attending the clinics.

### Conceptual Framework of Variables and Measures

Risk modeling studies can benefit from the identification of a coherent conceptual framework at the outset of the analysis [12]. This project will adopt the proximate-determinant framework in the selection, operationalization, and interpretation of explanatory variables [13]. To help clarify the relative strength or importance of each STI predictor, we categorized the predictors into two groups based on the proximate-determinants framework: (1) distal determinants, which are demographic, social, or economic variables distally related to STIs, and (2) proximate determinants, which are directly associated with an individual’s probability of exposure to STIs and the efficiency of STI transmission (Table 1).

The proximate-determinants framework hypothesizes that after adjustment for the proximate determinants or sociobehavioral predictors, relationships between the underlying or sociodemographic characteristics should be nonsignificant [13]. Underlying determinants included in the analytical framework are-age, gender, race/ethnicity, sex work, drug use, and residence. Proximate determinants included indices of sexual activity (number of sexual partners in the past six months, number of lifetime sex partners) and partner characteristics (sex work, Internet partners). Other variables included condom use, previous STI diagnosis, type of sex (anal, oral, vaginal), and gender of sex partners. Statistical analysis in this study will take advantage of the multilevel structure outlined in the framework to understand estimates of the associations between the determinants and acquisition of STIs.

**Table 1:** Candidate predictor and outcome variables for STI prediction rule modeling.

Variable		Description	Type
**Underlying determinants**			
	Age	Age in years	Interval
	Gender	Female/male/transgender	Categorical
	Race/ethnicity	Unspecified Aboriginal/Arab/Asian/Black/First Nations/Hispanic/Inuit/Metis/South Asian/White	Categorical
	Postal code	Self-reported postal code of residence	Categorical
	Street involvement	Self-reported street involvement–reported as yes/no	Dichotomous
	Sex trade worker	Self-reported sex trade worker–reported as yes/no	Dichotomous
	IDU^a^	Self-reported current or previous injection drug use–reported as yes/no	Dichotomous
**Proximate determinants**			
	Gender of sex partners	Female/male/both	Categorical
	Number of sex partners 6 months	Number of sexual partners in previous 6 months	Interval
	Number of sex partners lifetime	Number of sexual partners in lifetime	Interval
	Sex partner of IDU^a^	Yes/no/unknown	Categorical
	Type of sex	Anatomical sites reported by client as exposed to partner during recent sexual activity–reported as genital/insertive rectal/receptive rectal/throat	Categorical
	Number of Internet partners	Number of sexual partners met on Internet sites in previous 6 months	Interval
	Needle sharing	Self-reported sharing of needles for injection drug use–reported as yes/no	Dichotomous
	Sex partner of sex worker	Yes/no/unknown	Categorical
	Prior STI	Yes/no	Dichotomous
	Condom use	Self-reported condom use–reported as yes/no/sometimes	Categorical
**Health outcomes**			
	Chlamydia test result	Positive versus negative based on NAAT^b^ result	Dichotomous
	Gonorrhea test result	Positive versus negative based on NAAT^b^ result	Dichotomous

^a^Injection drug use

^b^nucleic acid amplification test

### Outcome Variables

The outcomes measured in this study will be diagnosis with chlamydia and/or gonorrhea infection [14]. Practitioners at sexual health clinics may order the following specimens-urine specimens and swabs (cervical, vaginal, urethral, rectal, oral swabs), which are tested using the nucleic acid amplification test (NAAT) or culture (gonorrhea only) [14]. We chose to examine chlamydia and/or gonorrhea as a composite outcome because most laboratories use multiplex assays that test for both infections simultaneously [15].

### Data Quality

The presence of missing data is a frequently encountered problem in the derivation and validation of prediction rules [16]. The default strategy is to delete all incomplete observations from the analysis; however, this is often a precarious and wasteful approach as variables are rarely missing at random. In this study, variables such as race/ethnicity, condom use, and number of sexual partners in previous 6 months have rates of missingness ranging from 8.92% (n=36,107) to 42.24% (n=36,107). Imputation techniques, especially multiple imputations, have been increasingly advocated to address the issue of missing values [17]. This study will impute missing values using IVEware, a software application that performs multiple imputations of missing values using the Sequential Regression Imputation Method [18]. In this method, imputations for each missing variable are produced based on a regression model using other variables as predictors in a cyclic manner [19].

### Sample Size

In prediction modeling, statistical precision is dependent on the number of individuals who experience the outcome of interest. Some authors have recommended that at least 10 individuals having the outcome of interest are needed per variable to allow for accurate prediction modeling (ie, events per variable-EPV) [11,17]. In this study, the derivation, temporal and geographical validation populations are sufficiently powered, having 11, 20, and 33 EPV, respectively.

### Analytic Plan

Descriptive statistics will be used to determine the frequency and distribution of each independent variable. All data analyses will be conducted using SAS v9.3. The primary outcome is chlamydia and or/gonorrhea diagnosis between 2000 and 2012. Continuous variables will be categorized based on clinically and epidemiologically relevant cutoff points [9]. The association between each predictor and the outcome will be examined using unadjusted prevalence odds ratios with the associated 95% confidence intervals. A stepwise technique will be used with variables selected for inclusion in the model on the basis of a significant change in the log likelihood (*P*<.05). We will initially explore separate models for males and females.

A score will be calculated by multiplying the regression coefficients of each variable by 5 in the final regression model, with rounding to simplify the calculation. These scores are an immediate reflection of the logarithm of the odds of infection [4]; they will be added into a sum score for each individual. To identify an optimal strategy to identify STI cases, a cutoff for the predicted probability will be calculated. Patients with predictions above the cutoff will be classified as positive; those under the cutoff as negative. Specifically, the performance of the prediction rule will be assessed on the basis of cases detected (sensitivity) and the number of clients who have been tested (efficiency) [20]. We will explore optimal risk score cutoff points that identify the most cases (a high percentage for sensitivity), while testing the fewest number of people (low percentage for efficiency) [6,21].

### Assessment of Model Performance Measures

We will explore two measures of model accuracy-calibration and discrimination. Calibration (or “reliability”) refers to the agreement of predicted and observed predictions [22]. Calibration will be tested using the Hosmer-Lemeshow goodness-of-fit test [20]. This test divides individuals into groups based on percentiles of their predicted probabilities of having an infection and then calculates within each group the expected number of positive and negative individuals [22]. These will then be compared with the observed values for the groups and the Pearson chi-squared statistic will be used to test for differences; *P*<.05 casts doubt on the fit of the model [20]. Calibration will also be assessed graphically by plotting observed frequencies of infection against predicted probabilities by a decile of predictions, drawing a line of regression through the points, and assessing the calibration slope [23]. The ideal calibration slope of a well-discriminating model is 1 [23].

Discrimination refers to the model’s ability to distinguish low risk from high risk individuals [6]. Discrimination will be quantified by the area under the receiver operating characteristic curve (AUC) or the *c-*statistic which will be constructed by graphing sensitivity against 1-specificity for different cutoff points of the predicted STI risk [4]. The AUC lies between zero and one and provides a measure of the ability of the model to discriminate between those who have an STI diagnosis and those who do not. A value of 0.5 suggests no discrimination, such that the model is no better than a random guess, whereas a value of 1.0 suggests perfect discrimination [20].

### Evaluation of Internal Validity

Internal validation is important to obtain an honest estimate of performance for patients that are similar to those in the training sample. Also, internal validation indicates an upper limit to the expected performance in other settings [23]. Evaluating the performance of the model on the same data used to create the model usually leads to an optimistically biased assessment or overfitting [23]. We will use bootstrap validation techniques to correct for the optimism bias. Random bootstrap samples will be drawn with replacement from the full sample (200 replications) and the performance of the developed model will be tested in similar populations as the derivation population [24]. This method will be used to estimate the overoptimism of the derived model and to, subsequently, adjust the measures of performance and the estimated regression coefficients in the final model for overfitting [17,24].

### Examining the External Validity of the Prediction Rule in Other Populations

Even when internal validation methods are used to correct for overfitting and optimism, the accuracy of prediction rules can be considerably lower in new populations compared to the accuracy found in the derivation population [25]. External validation is a stronger test of model performance, and will be determined in other populations that are plausibly related to the derivation population. We will assess the performance of the prediction rule in different temporal and geographical settings; these settings may be different from the derivation population due to, for example, variation in prevalence, diagnostic tests, access to sexual health care services, core groups, or sexual networks. These validation settings may also differ due to documentation or charting practices. The predicted probability for STI diagnosis in the *validation populations* will be calculated according to the previously calculated risk scores. The discriminative ability of the risk scores will be assessed by calculating the AUC and conducting the Hosmer-Lemeshow test as described above [6].

When the accuracy of the prediction rule in the *validation population* is poor, researchers often discard the rule and directly pursue deriving new rules with the data of the *validation population* only [25]. In this scenario, when every new setting leads to a new prediction rule, prior information captured in previously derived prediction rules would be neglected; this is counterintuitive as scientific inferences should be based on data of as many individuals as possible and also violates the scientific principle of updating prior knowledge from previous studies [25]. Several approaches for updating previously developed rules have been suggested in the literature [23,25]. In this study, we anticipate that due to the higher prevalence of infection in the validation datasets, the calibration of the rule in the *validation populations* may be poor as a result of systematically too low predicted probabilities [26]. The intercept, which reflects the risk of the outcome not explained by the predictors in the prediction model, will be adjusted such that the mean predicted risk equals the observed prevalence in the STI clinics outside of Vancouver [26,27]; thus, in updating the intercept, potentially poor calibration will be improved [26].

### Ethics

Ethics approval for the proposed thesis project has been obtained from the University of British Columbia Research Ethics Board prior to the start of any research activities. The BCCDC clinic data will be captured from existing program databases and will be considered chart review, which do not require informed consent. The stewards of these databases are BCCDC staff and the data will be safeguarded according to the Freedom of Information and Privacy Act.

## Results

This is an ongoing research project with initial results expected in 2014.

## Discussion

### Implications for the Scaling Up of Get Checked Online

The results from this research will have important implications for scaling up of Internet-based testing in BC. This analysis will focus on the development of screening criteria for asymptomatic heterosexuals, a population often targeted by screening recommendations issued by public health organizations. Several organizations recommend the screening of all sexually active men and women 25 years or younger for chlamydia. This recommendation could prove to be cost prohibitive in settings where individuals in this age group comprise the highest proportion of clinic visits and in low prevalence settings, whose STI epidemic could be characterized as concentrated, with low prevalence rates in the general population [4]. For example, there are increasing concerns about this recommendation, particularly in the United Kingdom, which has fully endorsed and committed funding to universal screening of young people (16 to 24 years) in the form of the National Chlamydia Screening Programme, an initiative reported to be facing implementation obstacles, low participation rates, and lack of demonstrable cost effectiveness [28].

### Improving on Screening Recommendations

Screening recommendations could be improved by tailoring risk assessment to the specific circumstances of the patient [29]. If a prediction rule with good calibration, discrimination, and high sensitivity to detect infection is found during this project, the prediction rule could be programmed into GCO so that the program offers individualized testing recommendations to clients. Further, the prediction rule could be adapted into educational materials to inform other Web-based content by creating awareness about STI risk factors which may stimulate health care seeking behavior among individuals accessing the website. For example, a potential use could be the creation of a Web-based risk assessment tool for individuals. The risk scores developed for the prediction rule also have important implications for risk communication and testing motivation because they can increase risk perception by creating tailored risk messages to different groups.

We also anticipate the prediction rule could potentially facilitate decision-making in traditional clinical encounters where clinicians could enter basic demographic and behavioral data directly into the client’s computerized medical record during the consultation. The prediction rule could be used to display an alert on the computer screen to prompt clinicians to offer specific STI tests to those at increased risk of infection. This would standardize both STI Web-based testing and at the clinics, ensuring those at greatest risk are tested and reduce unnecessary testing. Moreover, the results will be used to inform ongoing clinical recommendations related to selective screening of STI clients in BC, potentially enabling targeted testing to higher risk individuals, thereby reducing the unnecessary testing of those without the infection and saving costs.

### Study Strengths and Limitations

This study has several strengths. To our knowledge, this will be the first study to derive and validate a locally specific risk assessment tool to quantify STI risk in a Canadian setting. Risk assessment tools ideally should be derived from large representative samples [30]. Our study will include 13 years of electronic health records comprising more than 35,000 clinical visits to publicly funded STI clinics in BC, representing a high proportion of the population of individuals using this service in the province. One major limitation of this research project is that the validity of the predictive variables depends on the accuracy of the self-reported health behaviors. There is a risk of recall and social desirability biases because of the self-reported nature of stigmatized activities and behaviors. There is also the risk of overreporting of perceived normative behaviors such as condom use. Such reporting biases would artificially inflate the relationships between infection and risk factors. However, because the clinical risk assessment interviews are confidential and are conducted by clinicians who are typically not acquainted with the respondents, strong motivations to self-present are unlikely. Moreover, the outcome variables do not rely on self-report and thus are not subject to recall or social desirability biases. Another limitation is the limited generalizability of our prediction rule to the general population or people seeking care in settings other than STI clinics.

The ultimate goal of the proposed project is to use research evidence to inform policy and program development, and through providing more effective services, strengthen sexual health care provision in BC, including the optimal scaling up of GCO across BC (including in rural and northern communities). The investigators involved in this project are members of two GCO working groups; and, thus we are in positions to integrate knowledge translation as the data analyses progress and we examine preliminary findings [31]. The scholarly products developed as a result of the study (eg, manuscripts submitted to peer-reviewed journals, including open-access journals, presentations at conferences) will make theoretical and empirical contributions toward more effectively using the characteristics of sexual health clinic clients to predict STI.

## Multimedia Appendix 1

Canadian Institutes of Health Research decision letters for the project as well as the reviewer comments.

## Abbreviations

BC: British Columbia
BCCDC: BC Center for Disease Control
EPV: events per variable
GCO: Get Checked Online
HIV: human immunodeficiency virus
NAAT: nucleic acid amplification test
STI: sexually transmitted infections
