# Supplementary material for: Risk Prediction in Sexual Health Contexts: Protocol
Source: JMIR Res Protoc. 2013 Dec 3;2(2):e57. doi: 10.2196/resprot.2971 (PMC3868985; doi:10.2196/resprot.2971)
Supplement: Supplementary file 1 [file resprot_v2i2e57_app1.pdf]

CIHR IRSC

Institute of Aboriginal Peoples' Health
Institute of Aging
Institute of Cancer Research
Institute of Circulatory and Respiratory Health
Institute of Gender and Health
Institute of Genetics
Institute of Health Services and Policy Research
Institute of Human Development and Child and Youth Health
Institute of Infection and Immunity
Institute of Musculoskeletal Health and Arthritis
Institute of Neurosciences, Mental Health and Addiction
Institute of Nutrition, Metabolism and Diabetes
Institute of Population and Public Health

Institut de la santé des Autochtones
Institut du vieillissement
Institut du cancer
Institut de la santé circulatoire et respiratoire
Institut de la santé des femmes et des hommes
Institut de génétique
Institut des services et des politiques de la santé
Institut du développement et de la santé des enfants et des adolescents
Institut des maladies infectieuses et immunitaires
Institut de l'appareil locomoteur et de l'arthrite
Institut des neurosciences, de la santé mentale et des toxicomanies
Institut de la nutrition, du métabolisme et du diabète
Institut de la santé publique et des populations

April 16, 2012

Mrs. Titilola FALASINNU
University of British Columbia
School of Population and Public Health
2206 East Mall
Room 416
Vancouver, British Columbia V6T 1Z3

Dear Mrs. FALASINNU:

We are pleased to inform you that your recent application to the Canadian Institutes of Health Research (CIHR) for a Doctoral Research Award - PA: Patient-Oriented Research - Biostatistics has been approved.

An Offer of award, a Notice of Decision, review documents related to your application and a Response to an Offer of Award form are all available through your ResearchNet account. It is your responsibility to print and complete the Response to an Offer and mail it to CIHR within 15 business days of notification of your award.

Congratulations on your success in this competition. Should you have any questions, please do not hesitate to contact the Program Delivery Coordinator at: PA-AP@cihr-irsc.gc.ca

Sincerely,

NJM MacLellan

Nancy Mason MacLellan
Deputy Director, Program Delivery
Targeted Initiatives Branch
Research Portfolio

c.c.
SHOVELLER, Jean A

CIHR Office of Patient-Oriented Research

297521-201110DBI-DRB-266049-205326-PADPO

CIHR IRSC

Institute of Aboriginal Peoples' Health
Institute of Aging
Institute of Cancer Research
Institute of Circulatory and Respiratory Health
Institute of Gender and Health
Institute of Genetics
Institute of Health Services and Policy Research
Institute of Human Development and Child and Youth Health
Institute of Infection and Immunity
Institute of Musculoskeletal Health and Arthritis
Institute of Neurosciences, Mental Health and Addiction
Institute of Nutrition, Metabolism and Diabetes
Institute of Population and Public Health

Institut de la santé des Autochtones
Institut du vieillissement
Institut du cancer
Institut de la santé circulatoire et respiratoire
Institut de la santé des femmes et des hommes
Institut de génétique
Institut des services et des politiques de la santé
Institut du développement et de la santé des enfants et des adolescents
Institut des maladies infectieuses et immunitaires
Institut de l'appareil locomoteur et de l'arthrite
Institut des neurosciences, de la santé mentale et des toxicomanies
Institut de la nutrition, du métabolisme et du diabète
Institut de la santé publique et des populations

April 16, 2012

Mrs. Titilola FALASINNU
University of British Columbia
School of Population and Public Health
2206 East Mall
Room 416
Vancouver, British Columbia V6T 1Z3

Dear Mrs. FALASINNU:

Congratulations on your success in the recent Canadian Institutes of Health Research funding competition. You should take great pride in your success, particularly in light of the very competitive nature of CIHR peer review.

As you know, peer review is the cornerstone of our research funding system. This process rests on the kind voluntarism of your colleagues at other institutions who generously gave their time to review your application.

The Canadian Institutes of Health Research is committed to building an innovative national health research enterprise. To this end we have undertaken the development of a renewed strategic plan for CIHR, our Health Research Roadmap which has required support from researchers, policy makers, the voluntary sector and the Canadian public. To meet CIHR goals, we must share our knowledge. That is why we encourage you to work with your institution to communicate to Canadians about the work you are doing. To simplify this process, we have developed guidelines on public communication which you can find on our website at http://www.cihr-irsc.gc.ca/e/30789.html.

Once again, congratulations and I wish you success in your research.

Yours sincerely,

Alain Beaudet, MD, Ph.D.
President

President

Canadian Institutes of Health Research
Room 97, 160 Elgin Steet, Address locator: 4809A
Ottawa, (Ontario) K1A 0W9 Tel.: (613) 941-2672
Fax (613) 954-1800 **www.cihr-irsc.gc.ca**

Président

Instituts de recherche en santé du Canada
Pièce 97, 160 rue Elgin, Indice de l'adresse: 4809A
Ottawa, (Ontario) K1A 0W9 Tél.: (613) 941-2672
Fax (613) 954-1800 **www.irsc-cihr.gc.ca**

Canada

Application Number / Numéro de demande: 264970
Name of Applicant / Nom du candidat: FALASINNU, Titilola
Review Type / Type d'évaluation: Committee Member 1/Membre de comité 1
Competition: 2011-10-17 CIHR Doctoral Research Award
Concours: 2011-10-17 Bourse de recherche au doctorat des IRSC
Committee: Doctoral Research Awards B
Comité: Bourses de recherche au doctorat B

Assessment / Évaluation:

Achievements and Activities of the Candidate	Raw Score	Max Scores	Multiplier	Weighted Scores
1. Publication Activity	4.2	4.9	0.1	0.42
2. Other Research Activity	4.1	4.9	0.1	0.41
3. Academic Performance	4.0	4.9	0.15	0.60

Characteristics and Abilities of the Candidate	Raw Score	Max Scores	Multiplier	Weighted Scores
4. Reviewer Score	4.4	4.9	0.4	1.76

Research Training Environment	Raw Score	Max Scores	Multiplier	Weighted Scores
5. Training Program for the Candidate	4.2	4.9	0.1	0.42
6. Scientific Activity	4.0	4.9	0.05	0.20
7. Research Resources	4.4	4.9	0.05	0.22
8. Training Record	4.4	4.9	0.05	0.22

	Raw Score	Max Scores	Weighted Scores
**Total**	**33.70**	**39.2**	**4.25**

Application Number / Numéro de demande: 264970
Name of Applicant / Nom du candidat: FALASINNU, Titilola
Review Type / Type d'évaluation: Committee Member 1/Membre de comité 1
Competition: 2011-10-17 CIHR Doctoral Research Award
Concours: 2011-10-17 Bourse de recherche au doctorat des IRSC
Committee: Doctoral Research Awards B
Comité: Bourses de recherche au doctorat B

Comments:

excellent relevant experience
great project
program well suited for candidate's needs

Application Number / Numéro de demande: 264970
Name of Applicant / Nom du candidat: FALASINNU, Titilola
Review Type / Type d'évaluation: Committee Member 2/Membre de comité 2
Competition: 2011-10-17 CIHR Doctoral Research Award
Concours: 2011-10-17 Bourse de recherche au doctorat des IRSC
Committee: Doctoral Research Awards B
Comité: Bourses de recherche au doctorat B

Assessment / Évaluation:

Achievements and Activities of the Candidate	Raw Score	Max Scores	Multiplier	Weighted Scores
1. Publication Activity	3.9	4.9	0.1	0.39
2. Other Research Activity	3.9	4.9	0.1	0.39
3. Academic Performance	3.9	4.9	0.15	0.59

Characteristics and Abilities of the Candidate	Raw Score	Max Scores	Multiplier	Weighted Scores
4. Reviewer Score	3.9	4.9	0.4	1.56

Research Training Environment	Raw Score	Max Scores	Multiplier	Weighted Scores
5. Training Program for the Candidate	4.2	4.9	0.1	0.42
6. Scientific Activity	3.8	4.9	0.05	0.19
7. Research Resources	4.0	4.9	0.05	0.20
8. Training Record	4.0	4.9	0.05	0.20

	Raw Score	Max Scores	Weighted Scores
**Total**	**31.60**	**39.2**	**3.94**

Application Number / Numéro de demande: 264970
Name of Applicant / Nom du candidat: FALASINNU, Titilola
Review Type / Type d'évaluation: Committee Member 2/Membre de comité 2
Competition: 2011-10-17 CIHR Doctoral Research Award
Concours: 2011-10-17 Bourse de recherche au doctorat des IRSC
Committee: Doctoral Research Awards B
Comité: Bourses de recherche au doctorat B

Comments:

The proposed research has potential to provide important knowledge that can be used for improving health, and also dealing with the pandemic STDs. The research capacity and environment is adequate, and the candidate shows confidence in achieving the objectives of the research.
